# Genotoxic effects of tobacco use in residents of hilly areas and foot hills of Western Ghats, Southern India

**DOI:** 10.1038/s41598-019-51275-w

**Published:** 2019-10-17

**Authors:** R. Chandirasekar, K. Murugan, T. Muralisankar, V. Uthayakumar, R. Jayakumar, K. Mohan, C. Vasugi, R. Mathivanan, S. Mekala, A. Jagateesh, K. Suresh

**Affiliations:** 1 Human Molecular Genetics Laboratory, PG and Research Department of Zoology, Sri Vasavi College, Erode, 638316 Tamilnadu India; 2 Department of Zoology, Bharathiar University, Coimbatore, 641046 Tamilnadu India; 3 Department of Molecular Medicine, Faculty of Medicine, University of Malaya, Kuala Lumpur, 50603 Malaysia; 4 PG & Research Department of Zoology Chikkaiah Naicker College, 638004, Erode, Tamilnadu India; 5 Centre of Advanced Study in Marine Biology, Faculty of Marine Science, Annamalai University, Parangipettai, Tamil nadu 608 502 India; 6 Thiruvalluvar University, Serkkadu, Vellore, 632 115 India

**Keywords:** Cytogenetics, Population screening, Risk factors

## Abstract

Smoking and smokeless tobacco consumption is a significant risk factor that provokes genetic alterations. The present investigation was to evaluate the biomarkers of genotoxicity including micronucleus (MN), chromosome aberrations (CA) and DNA strand breaks among tobacco consumers and control individuals residing in hilly areas of Western Ghats, Tamilnadu, South India. This study included 268 tobacco consumers with equal number of controls. The tobacco consumers were divided into Group I (<10 years of tobacco consumption with an age range from 15 to 35 years) and group II (>10 years consumption above 35 years of age). Chromosome aberration (CA) and comet assay were performed using blood and micronucleus assay from exfoliated buccal epithelial cells obtained from tobacco consumers and controls. Elevated levels of CA were found in group II (Chromatid type: 2.39 ± 1.13 and chromosome type: 1.44 ± 1.24) exposed subjects, high micronucleus and DNA damage (TL:4.48 ± 1.24 and TM:3.40 ± 1.58) levels were significantly (*p* < 0.05) observed in both smoking and smokeless tobacco consumers when comparison with group I and controls. This study also observed a lack of awareness among the tobacco consumers about the harmful health effects of tobacco. Tobacco consumption contributes to the significant alteration in genetic materials. In addition, a high rate of spontaneous abortion was also seen in the studied population.

## Introduction

The global burden of tobacco use is borne by low-and middle-income countries, particularly the countries in Asian continent. China is the biggest tobacco marketer in the world, with more than 300 million smokers, followed by India, with over 100 million^1^.

Smoking tobacco products are considered as an important issue in public health as it promotes oxidative stress that play important role in inducing many diseases^2^. Consumption of smokeless tobacco (SLT) are common in many parts of the world and the rate is higher in India^3^. A study on SLT reports that more than 100 million people are consuming tobacco related products in India and Pakistan^4^. Epidemiological reports correlated SLT usage with high incidences of oral cancer^5^. It is reported that the prevalence of oral cancer are elevated in countries where tobacco was habitually consumed along with betel quid. SLT has been evaluated and classified as ‘Carcinogenic to Humans’^6^. Oral leukoplakia is the majority repeated premalignant lesion with prevalence ranging from 0.2% to 5% and has a cumulative risk of malignant transformation ranging from 0% to 38% and an average annual transformation rate of 1%^7^.

The important risk factor that induces alterations in genetic material is found to be the consumption of tobacco products^8,9^. Tobacco smoking is the causative agent for cancer of the lung, larynx, oral cavity, bladder, pancreas, cervix, kidney, stomach, blood, liver, colon and rectum, and esophagus and also induces the risk of diabetes and rheumatoid arthritis^10^. Tobacco smoke contains more than 5,000 chemicals^11^.

Chromosome aberration analysis has been commonly performed on human peripheral blood lymphocytes to assess DNA damage^12^. The micronucleus (MN) and comet assay are used to assess the genotoxic effects of tobacco products among the consumers who are at a high risk in developing cancer^13,14^. CAs and MN frequency have been proposed as sensitive parameters for assessing genotoxic effects of chemical or physical mutagens^15^. In addition Single-cell gel electrophoresis (SCGE) or comet assay is also used to monitor genotoxicity in the exposed population. It detects different kinds of DNA alterations and single strand breaks^16^.

The prime aim of this investigation was to analyze the extent of genotoxic damage induced by tobacco products in tribal people through chromosome aberration, micronucleus and comet assays.

## Materials and Methods

The study was conducted on a total of 268 people living in hilly areas and foot hills of Western Ghats (Bargur, Thamaraikkarai, Oosimalai). The study subjects were classified based on the consumption of tobacco as smoking (Beedi and Cigarettes) and SLT (betel quid, areca nut and tobacco leaf) consumers and an equivalent number of healthy normal controls (268) were recruited with matched age (±5) and gender were included in the study. Ethical approval for the study was obtained from Medical Review Committee, Dhanvantri College of Nursing, Tiruchengode, Namakkal, Tamilnadu. A written informed consent (Supplementary file) was obtained from all the study participants before collecting blood and buccal epithelial cells. Information regarding the exposure duration, types of tobacco usage, other health complaints (Spontaneous abortion, Hypertension, Diabetes and Cardiac problems) and occupation was recorded using questionnaire of the study participants, 103 were males and 165 were females. Further, the tobacco consumers were categorized into two groups such as group I and group II based on the age and duration of tobacco consumption. Group I (<10 years of tobacco consumption) with an age range from 15 to 35 years and group II (>10 years of tobacco consumption) above 35 years of age. About 3–5 ml of venous blood was collected in a heparin coated vacutainers for the analysis of CAs, MN and DNA damage using single-cell gel electrophoresis (SCGE) comet assay. For MN assay, buccal epithelial cells were collected by scraping buccal mucosa.

### Analysis of micronucleus in peripheral blood lymphocytes

Cytokinesis blocked micronucleus assay was carried out as described by Fenech and Morley^17^. In brief, 0.5 mL of blood was added to 4.5 mL of RPMI-1640 medium (Hyclone) enriched with 20% FBS (Gibco), 2 mM L-Glutamine (Gibco) and 0.2 mL of phytohemagglutinin (Gibco). The mixture was incubated at 37 °C for 72 hours. Cytokinesis was blocked at 44 hrs of incubation by the addition of cytochalasin B to cells at a final concentration of 6 µg/mL. After 72 hrs of incubation, cells were harvested and treated with 5 ml of hypotonic solution (0.075 M KCl) for 15 minutes and fixed in fresh fixative solution (Methanol: Acetic acid, 3:1). This fixation step was repeated twice after 20 minutes of storage at 4 °C and eventually, stained in giemsa stain. A total of 1000 cells were scored.

### Study of micronucleus in buccal cells

After rinsing the mouth in water, buccal epithelial cells were collected by scraping the right and left cheek mucosa with spatula. Cells were transferred to saline solution (0.9% NaCl). The cells were centrifuged (800 rpm) for 5 minutes, fixed in 3:1 methanol/acetic acid, and dropped onto cleaned slides. The slides were stained in Feulgen plus fast green solution. The identification of micronucleus was based on the criteria proposed by Sarto *et al*.^18^. About 2,000 cells were screened for calculating the frequency of micronucleated cells in each study participant.

### Study of chromosome aberrations

Peripheral blood lymphocytes were cultured following a standard procedure^19^. Briefly, 0.5 mL of whole blood was added to 4.5 mL of RPMI 1640 medium supplement with 15% of FBS, 2 mM of L-glutamine, 100 U/mL of penicillin and 100 µg/mL streptomycin and 0.2 mL of phytohemagglutinin (PHA). The whole culture was incubated at 37 °C for 72 hrs and the culture tube received periodical shaking twice a day to aid proper mixing of the medium and cells. After 71 hrs, cultures were treated with 0.01 mg/mL colcemid to arrest the cells at mitotic stage. Cells were harvested after 72 hrs by centrifuging to eliminate culture medium (800–1000 rpm), then warmed (37 °C) hypotonic solution (KCl 0.075 M) was added and treated for 20 min. The cells were treated with Carnoy’s fixative (3:1 ratio of methanol: acetic acid). Slides were prepared and carefully dried. Later, slides were stained using giemsa stain. The metaphase chromosomes were viewed under light microscope with 100X magnification. About 100 metaphase spreads were selected and analyzed for each sample. Our study analyzed the aberrations such as dicentrics, ring, gaps and breaks.

### Analysis of DNA damage by single cell gel electrophoresis

Comet assay was performed as illustrated by Tice *et al*.^20^. Briefly, the blood cells were lysed in lysis solution (2.5 M NaCl, 10 mM Tris, 100 mM EDTA and 1% C_3_H_6_NNaO_2_ and *p*H 10) at 4 °C for 48 hrs and dropped in a freshly prepared lysis solution containing 140 µL of proteinase K at 37 °C for 2 hrs.

Slides were placed on horizontal gel electrophoresis unit chamber. The DNA was allowed to unwind for 20 min in electrophoresis running buffer solution (300 mM NaOH and 1 mM Na, EDTA, *p*H 13). Electrophoresis was conducted for 20 min at 25 V and 300 mA. Than the slides were removed and the alkaline *p*H was neutralized with 0.4 M Tris-HCl, *p*H 7.5. EtBr (75 µL of 20 mg/mL) solution was added to each slides and the cover glass was placed over the gel. DNA damage was quantified by visual categorization of cells into grouping of comets consequent to the amount of DNA material present in the tail. 100 images were randomly selected and analyzed for both individuals. Comet tail length (nuclear region + tail) was calculated in random units. The fluorescence microscope (Labomed) was prepared with a BP546/12-nm excitation 590-nm barrier filter. Tail length (TL) and tail moment (TM) was assessed. TL (length of DNA migration) was related directly to the DNA fragment size and existing in micrometers. Tail moment was calculated as the invention of the TL and the portion of DNA in the tail.

### Statistical analysis

Statistical examination was carried out using the statistical software (SPSS Version 16). Analysis of variance (one way ANOVA) was carrying out to match up to the frequency of CAs, MN and DNA damage by comet between tobacco consumers and controls. *p* < 0.05 was considered as a significance level.

### Human blood samples collection statement

We confirmed that all experiments were performed in accordance with relevant guidelines and regulations.

The study was approved by the Medical Review Committee, Dhanvantri College of Nursing, Tiruchengode, Namakkal, Tamilnadu and questionnaire collected and informed written consent was obtained from all the study participants before collecting blood and buccal epithelial cells. Great care was taken not to harm subjects and appropriate medical procedures were followed during blood collection by a skilled medical nurse. The work was followed and carried out in accordance with the guidelines/regulations and ethical standards laid down in the 1964 Declaration of Helsinki.

## Result

A total of 268 participants were enrolled in this study, among them 103 were males and 165 were females. According to the year of exposure to tobacco and age, the participants were further classified in to two groups (group I and group II). Among the demographic data tobacco exposures were higher in female subjects in particularly, Smokeless tobacco usage was found to be high in females than in males. From the questionnaire it was observed that the participants reported diseases including cardiovascular, diabetes mellitus, hypertension and spontaneous abortions (Table 1).

**Table 1:** Demographic details of age and year of exposure and risk factors in tobacco users.

Particulars	No. of sample and %	Year of exposure Mean ± SD	Age Mean ± SD	BP (%) (Blood Pressure) RR :120/80	Diabetes (%)	Spontaneous Abortion	Cardiac Complaint
**Total Experimentals**	268	11.74 ± 5.97	43.70 ± 15.78	36 (13.43)	41 (15.30)	16 (5.97)	8 (2.98)
Male	103 (38.43)	12.74 ± 6.94	42.55 ± 16.23	17 (16.50)	18 (17.47)		6 (5.82)
Female	165 (61.56)	11.11 ± 5.21	44.41 ± 15.50	19 (11.51)	23 (13.93)	16 (5.97)	2 (1.21)
**Controls**							
**Group I**	112 (41.79)	—	32.08 ± 8.57	—	1 (0.97)		—
Male	39 (34.81)	—	29.74 ± 6.79	—	—	—	—
Female	73 (65.17)	—	33.34 ± 9.19	—	1 (1.36)	—	—
**Group II**	156 (58.20)	—	53.33 ± 8.58	5 (3.20)	5 (8.33)	—	—
Male	64 (41.02)	—	54.25 ± 9.32	3 (4.64)	2 (3.12)	—	—
Female	92 (58.97)	—	52.70 ± 8.01	2 (2.17)	3 (3.26)	—	—
**Experimental**						—	
**Group I**	112 (41.79)	6.26 ± 1.86	30.31 ± 6.50	4 (3.57)	8 (7.14)	—	—
Male	39 (34.81)	5.69 ± 2.02	27.80 ± 6.74	1 (2.56)	3 (7.69)		—
Female	73 (65.17)	6.57 ± 1.71	31.65 ± 5.98	3 (4.510)	5 (6.84)	16 (5.97)	—
**Group II**	156 (58.20)	15.67 ± 4.67	53.54 ± 13.49	32 (20.51)*	33 (21.15)	—	—
Male	64 (41.02)	17.04 ± 5.10	51.54 ± 13.49	16 (25.00)	15 (23.43)	—	6 (5.82)
Female	92 (58.97)	14.71 ± 4.11	54.54 ± 13.05	16 (17.39)	18 (19.56)	—	2 (1.21)
**Between Experimental**	268						
Smokers	6 5(24.25)	10.96 ± 6.99	35.78 ± 14.91	1(1.53)	3(4.61)		—
SL tobacco users	154 (57.46)	11.03 ± 5.31	41.96 ± 14.18	20 (12.98)	22 (14.28)	16 (5.97)	—
Smokers and SLT users	49 (18.28)	14.97 ± 5.51	59.65 ± 09.65	35 (71.42)*	16(32.65)		8 (2.98)

SD = standard deviation. FS: Female subjects, RR: Reference range. *Significantly higher when compared to the other groups.

### Micronucleus frequency in blood and buccal epithelial cells

Peripheral blood MN level was significantly (*p* < 0.05) elevated in group I tobacco consumers (1.73 ± 0.90) when compared to the age and sex matched group I control (0.70 ± 0.65). Group II tobacco consumers also showed a significantly elevated level of blood micronucleus frequency (2.46 ± 0.88) when compared to group II control (1.16 ± 0.67). A comparative analysis on individuals having both smoking and smokeless tobacco habits showed high levels of MN frequency (2.61 ± 0.93) than the individual having either smoking (2.12 ± 0.83) or smokeless tobacco (2.02 ± 0.98) habits (Table 2).

**Table 2:** Frequencies of micronuclei scored in buccal and blood cells of tobacco users and controls.

Particulars	No. of subjects studied	Mean frequency of micronuclei ± standard deviation of MN scored in blood cells/1000 (mean ± SD)	Mean frequency of micronuclei ± standard deviation of MN scored in buccal cells/2000 (mean ± SD)	Blood *p* < 0.005	Buccal *p* < 0.005
**Controls**					
**Group I**	112	0.70 ± 0.65	0.76 ± 0.69		
Male	39	0.82 ± 0.68	0.69 ± 0.61	0.002	
Female	73	0.64 ± 0.63	0.80 ± 0.73		0.031
**Group II**	156	1.16 ± 0.67	1.08 ± 0.82	0.003	0.45
Male	64	1.25 ± 0.69	1.03 ± 0.77		
Female	92	1.10 ± 0.67	1.13 ± 0.85		0.001
**Experimentals**					
**Group I**	112	1.73 ± 0.90	2.19 ± 1.42*	0.004	
Male	39	1.87 ± 0.76	2.46 ± 1.41		0.001
Female	73	1.65 ± 0.96	2.05 ± 1.42		0.909
**Group II**	156	2.46 ± 0.88^#^	3.01 ± 1.38*		0.001
Male	64	2.65 ± 0.82**	2.96 ± 1.40	0.001	0.002
Female	92	2.32 ± 0.91	2.67 ± 1.46		
**Between Experimentals**	268				
Smokers	65	2.12 ± 0.83	2.90 ± 1.55		0.001
SLT users	154	2.02 ± 0.98	2.53 ± 1.34		
Smokers and SLT users	49	2.61 ± 0.93^a^	2.79 ± 1.64	0.001	0.001

^*,#^Significantly elevated when compared to controls subjects as estimated by ANOVA.

**Significantly elevated compared to controls and group I and female experimental subjects.

^a^Significantly elevated compared to controls and smokers experimental subject as estimated by ANOVA followed by Bonferroni’s correction for multiple comparisons.

**Figure 1:**
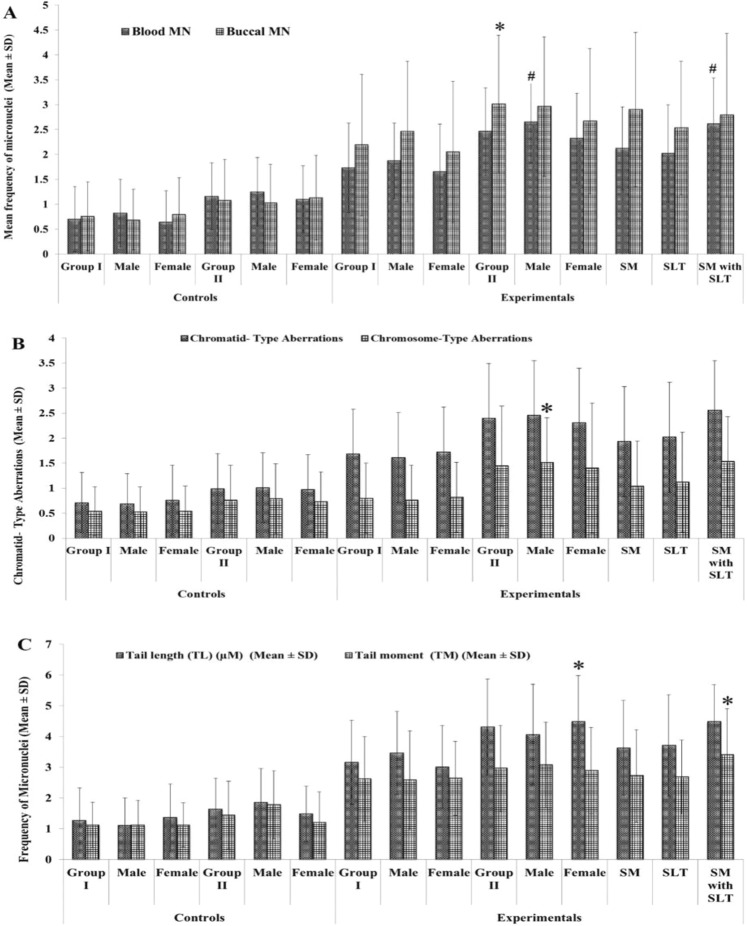
(**A**) Represent the frequencies of micronuclei in buccal and blood cells of tobacco exposures and controls. Blood MN level was elevated when compared to the controls and other subjects. ^#^indicates significantly higher when compared to female and other groups. (**B**) Shows the chromosome alterations in controls and experimental subjects. A super scribed (*) indicates the data is significance at *p* < 0.005 level when compared to other subjects. (**C**) Demonstration of comets scored in blood cells of tobacco users and controls. A super scribed (*) indicate the significance at *p* < 0.005 level when compared to other subjects.

Buccal epithelial cell MN frequency was significantly higher (*p* < 0.05) in both tobacco consumers group I (2.19 ± 1.42) and group II (3.01 ± 1.38) in comparison to their respective controls. A comparative analysis on individuals having both smoking and smokeless tobacco habits showed high levels of buccal MN frequency than the smoking or smokeless tobacco. In addition, our study compared the MN frequency in peripheral blood and buccal epithelial cell among group II males with group II females and the results showed that males of group II showed significantly (p < 0.05) elevated levels of MN frequency than the latter (Fig. 1A).

### Chromosome aberration analysis

A significantly increased number of gaps and breaks (Minor abnormalities) were observed in group I (1.68 ± 0.93) and group II (2.39 ± 1.13) tobacco consumers when compared with group I (0.71 ± 0.80) and group II (0.99 ± 0.71) controls respectively. Chromosome type aberrations such as dicentrics and rings were found to be present in higher numbers in group I (0.80 ± 0.75) and group II (1.44 ± 1.24) tobacco consumers than in group I (0.54 ± 0.51) and group II (0.76 ± 0.71) controls respectively (Fig. 1B). Among the tobacco consumers, individuals consume both smoke and smokeless tobacco habit subjects had higher levels of aberrations (Chromatid type −2.55 ± 1.19 and Chromosome type-1.53 ± 0.93) than individuals consume either smoke or smokeless tobacco (Table 3).

**Table 3:** Frequencies of chromosome aberrations in smokeless tobacco users and controls.

Subjects	Number of samples	Chromosome aberrations		Chromatid- type aberrations	Chromosome-type aberration
		Chromatid- type aberrations (mean ± SD)	Chromosome-type aberration (mean ± SD)	*p* < 0.005	*p* < 0.005
**Controls**					
**Group I**	112	0.71 ± 0.80	0.54 ± 0.51		
Male	39	0.69 ± 0.79	0.53 ± 0.50		
Female	73	0.76 ± 0.80	0.54 ± 0.52	0.324	1
**Group II**	156	0.99 ± 0.71	0.76 ± 0.71		
Male	64	1.01 ± 0.70	0.79 ± 0.80	0.052	0.912
Female	92	0.97 ± 0.72	0.73 ± 0.66		0.031
**Experimentals**					
**Group I**	112	1.68 ± 0.93	0.80 ± 0.75		
Male	39	1.61 ± 0.96	0.76 ± 0.80	0.001	
Female	73	1.72 ± 0.91	0.82 ± 0.73		0.325
**Group II**	156	2.39 ± 1.13^**#**^	1.44 ± 1.24		0.001
Male	64	2.45 ± 1.19	1.51 ± 0.99	0.001	0.235
Female	92	2.30 ± 1.10	1.40 ± 1.39		
**Between experimentals**	268				
Smokers	65	1.93 ± 1.13	1.04 ± 0.94		
SLT users	154	2.02 ± 1.04	1.12 ± 1.20		
Smokers and SLT users	49	2.55 ± 1.19*^a^	1.53 ± 0.93*^a^	0.001	0.001

^*^Significantly elevated when compared to the controls and smokers subjects as estimated by ANOVA.

^#^Significantly elevated compared to the group I subjects.

^a^Significantly elevated when compared to controls and smokers experimental subject as estimated by ANOVA followed by Bonferroni’s correction for multiple comparisons.

### DNA damage by Single cell gel electrophoresis

The DNA Tail length (TL) and Tail Moment (TM) was found to be significantly higher in tobacco consumers group I (TL: 3.16 ± 1.36 & TM: 2.62 ± 1.38) and group II (TL: 4.31 ± 1.56 and TM: 2.96 ± 1.41) and the extend of DNA damage was further increased in individuals with both habit (smoking and SLT usage) (TL: 4.48 ± 1.24 and TM: 3.40 ± 1.58) as compared to their respective controls (Table 4 and Fig. 1C).

**Table 4:** Frequencies of comets scored in blood cells of tobacco users and controls.

Subjects	Number of samples	Tail length (TL) (µM) mean ± SD)	Tail moment (TM) (mean ± SD)
**Group I**	112	1.27 ± 1.06	1.12 ± 0.74
Male	39	1.10 ± 0.91	1.12 ± 0.80
Female	73	1.36 ± 1.13	1.12 ± 0.72
**Group II**	156	1.64 ± 1.05	1.44 ± 1.15
Male	64	1.85 ± 1.11^#^	1.78 ± 1.17^a^
Female	92	1.48 ± 0.99	1.20 ± 1.09
**Experimentals**			
**Group I**	112	3.16 ± 1.36	2.62 ± 1.38
Male	39	3.46 ± 1.35	2.58 ± 1.61
Female	73	3.01 ± 1.35	2.64 ± 1.26
**Group II**	156	4.31 ± 1.56*	2.96 ± 1.41
Male	64	4.06 ± 1.63	3.07 ± 1.40
Female	92	4.48 ± 1.50^#^	2.89 ± 1.41
**Between Experimentals**	268		
Smokers	65	3.63 ± 1.54	2.72 ± 1.53
SLT users	154	3.71 ± 1.65	2.68 ± 1.25
Smokers and SLT users	49	4.48 ± 1.24**	3.40 ± 1.58

^*^p < 0.001 level significantly elevated compared to controls groups.

^#^Significantly elevated when compared to controls and group I experimental male and female subject.

^**^Significantly elevated when compared to controls and smokers and SLT experimental subject as estimated by ANOVA followed by Bonferroni’s correction for multiple comparisons.

## Discussion

All the genotoxic biomarkers revealed that the DNA damage was significantly increased in tobacco consumers than controls. Individuals with both smoking and smokeless usage harbored higher levels of DNA damage. The stuffing of tobacco have been recognized as mutagenic^21^. Tobacco usage has been inducing an assortment of genetic abnormality, including gene mutations, CA, MN and DNA strand breaks^22^. Lee *et al*.^23^ reported a positive association between chewing tobacco and oral cancers.

In this study, MN frequency (peripheral blood and buccal epithelial cells) was significantly increased in smokers and SLT users compared to the controls. This increase may be due to the presence of genotoxic agents in tobacco as reported previously^24^. Bonaasi *et al*.^25^ reported that, smokers had increased MN frequency than non smokers. Higher MN frequencies in buccal epithelial cells of SLT users were reported by Kausar *et al*.^26^. The study results are in concurrence with a previous study that reported increased MN frequency in smokers^27^. The MN test consequently used for early detection of carcinogenic process^28,29^. In addition, Kamboj and Mahajan^30^ reported that, abnormal tobacco oral habits significantly increase the frequency of MN. Our subsequent studies have point out that the buccal MN frequency was significantly higher in active and passive smokers compared to controls^31^. Orta and Gunebakan^32^ reported that, MN frequency increases with age of the subjects.

The common increase in tobacco chewing connected to oral cancers are on chromosomes 8p, 9p, 9q, 17q and 20q and the most common losses are in chromosome arms 3p, 4q, 5q, 9q and18q^33,34^. The higher CA in human lymphocytes forecast risk of cancer^35^ and also associated with MN frequency in lymphocytes^36^. Genomic damages are admirable biomarkers of exposure to chromosome-damaging products in tobacco. An elevated level of CA frequency shows 80% increased risk of cancer^37^. Active smokers posses higher level of CAs than the passive smokers^38^. Smoking habit as an imperative part that induces the significant alterations in the genomic material^8,9^.

In the present studies, CAs were scored in the blood lymphocytes of tobacco users and controls. The CA levels in group II (above the 35 years of age) was significantly higher than the controls. With respect to CAs, the aberration levels were significantly higher in both smokers and smokeless users compared to the smokers and controls. The period of exposure was higher in group II subjects than in group I subjects. This result suggested that age and duration of exposure might be a contributing factor for the increased CAs among tobacco users.

Fenech *et al*.^39^ suggested that chromosome damage increases with increasing age. In the present study CAs were analyzed in all the recruited individuals. The group II individuals have higher number of chromosome aberration compared to controls and group I due to their higher age and high level of tobacco exposure. Hence, age and tobacco consumed duration may play an important role in inducing chromosome aberration in the study population. Previous report stated that chromosomal damage is closely associated with age and smoking habits^9^. The MN and CA tests are considered as key genotoxic tests to assess the genotoxicity of chemicals^40^.

Recent study concludes that the smokers had the risk of genetic alteration^41^. The alkaline comet assay is a well established method used to measure the genetic toxicology^42^. Jayakumar and Sasikala^43^ reported that comet assay is extensively used for evaluation of DNA damage in occupational and environmental exposed populations. In the present study, comet assay was used to assess the DNA damage in tobacco exposure. The DNA damage was analyzed in the present study where the DNA Tail length and Tail Moment was scored in tobacco consumers and controls. The results showed that DNA migration was found to be significantly higher in the tobacco consumers (smoking and SLT usage) as compared to the control groups. Subjects who were above 35 years of age and more than 10 years of tobacco consumption showed a significant increase in DNA damage than the control.

## Conclusion

In conclusion, our result shows a significant elevated level of cytogenetic abnormalities among the tobacco users of both kinds. Age and duration of tobacco consumption are two major factors that induce higher degree of genotoxic effects among the consumers.

## Supplementary information

Supplementary Dataset 1-3
