# Supplementary material for: Genotoxic effects of tobacco use in residents of hilly areas and foot hills of Western Ghats, Southern India
Source: Sci Rep. 2019 Oct 17;9:14898. doi: 10.1038/s41598-019-51275-w (PMC6797791; doi:10.1038/s41598-019-51275-w)
Supplement: Supplementary file 1 — Supplementary Dataset 1-3 [file 41598_2019_51275_MOESM1_ESM.doc]

**Genotoxic effects of tobacco use in residents of hilly areas and foot hills of Western Ghats, Southern India**

R. Chandirasekar ^1*#^, K. Murugan ^2^, E. Anitha^1^, V. Uthayakumar^1^, R. Jayakumar^3#^, K. Mohan^4^,
R. Mathivanan^1^, M. Raghadevi^1^, K. Loganathan^1^, A. Jagateesh^5^, K.Suresh^6^.

^1^Human Molecular Genetics Laboratory, PG and Research Department of Zoology, Sri Vasavi College, Erode, India 638316.

^2^Thiruvalluvar University, Serkkadu, Vellore - 632 115 Tamilnadu, India.

^3^ Molecular Medicine Department, Faculty of Medicine, University of Malaya, Kuala Lumpur, Malaysia 50603.

^4^Centre of Advanced Study in Marine Biology, Faculty of Marine Science, Annamalai University, Parangipettai 608 502.

^5^PG & Research Department of Zoology Chikkaiah Naicker College, Erode, Tamil nadu-638 004

^6^Department of Zoology Bharathiar University, Coimbatore-641046.

^#^These authors contributed equally to this work

*Corresponding author:

**Dr. R.Chandirasekar,**

PG and Research Department of Zoology

Sri Vasavi College, Erode.

(Affiliated by Bharathiar University)

Tamilnadu, India-638316. Tel: +91-9942520304; E-mail: [genechandru@gmail.com](mailto:genechandru@gmail.com)

**(Patient ID) DST-SERB**

**Health Questionnaire**

This health questionnaire is provided for research purposes only. The questionnaire inquires the following information: gender, occupation, smoking states, disease condition, type of disease, food habit, and treatment type, disease durations and total health profile.

**A. IDENTIFICATION**

Date :

Name :

Parent Name :

Address (present) :

Permanent :

City: State: Pin code:

Phone: Birth date: Age:

Gender: Male: Female

**B. SMOKING HISTORY**

Do you smoke : yes__ no__

Yes if : beedi - cigarettes -

Please estimate the amount you smoke daily _______ per day.

     How many years have you been smoking? __ Years.

1What are some reasons for you to quit using tobacco?

2What do you think are your barriers to quitting tobacco use right now?

**C.** **SMOKELESS TOBACCO USAGE**

Year of Usage:

Type of product:

1) Do you smoke? YES NO If yes, how many?

**D. MEDICAL HISTORY**

Have you ever noticed that some kinds of chemicals make you?

1 Cough? YES NO

2 Wheezes? YES NO

3 short of breath? YES NO

4 Experience chest tightness? YES NO

Do you take any kind of medicine regularly? YES NO

If YES, what kind?

Have you ever had a heart attack or heart trouble? YES NO

Do you cough as much as three months out of the year? YES NO

     a) If YES, have you had this cough for more than two years? YES NO

     b) If YES, do you ever cough anything up from chest? YES NO

Do you have a feeling of smothering, unable to take a deep breath, or tightness in your chest? YES NO

D. Do you suffer from any of the following health conditions? Yes No

• Diabetes

• Heart or circulatory disorders

• Stomach or intestinal disorders

• Any condition which causes difficulties sleeping

• Chronic chest disorders, especially if night time symptoms are troublesome

• Any medical condition requiring medication to a strict timetable

• Any other health factors that might affect fitness at work.

**E. Please indicates below which chronic condition (s) you have:**

Asthma, Emphysema or COPD, Other lung disease, Type of lung disease, Heart disease,

I. How does this medication affect your ability to exercise or achieve your fitness goals?

1) Do you drink alcohol? YES NO If yes, how many glasses per week?__________

2) Describe your job:  sedentary  Active  physically demanding

3) Does your job require travel? YES NO

4) On a scale of 1-10, how would you rate your stress level (1= very low 10 = very high)?

**5)** List your 3 biggest sources of stress:

A b. c

**H. What treatments do you now have for this?**

1 No treatment

2 Medicines, tablets, or pills

5 Other (Specify) -------------

**I. Nutrition Related Questions**

On a scale of 1-10, how would you rate your Nutrition (1=very poor 10=excellent)?

2) How many times a day do you usually eat (including snacks)? _______________

35) Do you eat late at night?  Sometimes  Often  Never

7) How many glasses of water do you consume daily? _____________

8) Do you feel drops in your energy levels throughout the day? YES, NO If yes, when?______

**Declaration**

I hereby declare that all the information given in this form is true to the best of my knowledge. Further I give my consent to draw blood (about 5 ml) and the same can be used for the research purpose.

**Signature**
